
JOURNAL INFORMATION
==============================
NLM Title Abbreviation: Hum Brain Mapp
Journal ID: Hum Brain Mapp
NLM Journal ID: 9419065
Journal ID: HBM

Human Brain Mapping

ISSN: 1065-9471
EISSN: 1097-0193
Publisher: Wiley

ARTICLE INFORMATION
==============================
PMCID: PMC11474788
DOI: 10.1002/hbm.25930
Article ID: HBM25930
Article version: 1
Subjects: Issue Information, Issue Information

Editorial board ‐ TOC

Electronic publication date: 2023 Jul 24
Publication date: 2023 Aug 15
Volume: 44
Issue: 12
First page: 4423
Last page: 4425
Copyright: © 2023 The Authors. Human Brain Mapping published by Wiley Periodicals LLC.
License: This is an open access article under the terms of the http://creativecommons.org/licenses/by/4.0/ License, which permits use, distribution and reproduction in any medium, provided the original work is properly cited.
License URL: https://creativecommons.org/licenses/by/4.0/

Figure count: 0
Table count: 0
Page count: 7

source-schema-version-number: 2.0
cover-date: 15 August 2023
details-of-publishers-convertor: Converter:WILEY_ML3GV2_TO_JATSPMC version:6.4.9 mode:remove_FC converted:15.10.2024

==============================

### HUMAN BRAIN MAPPING

Open Access

Volume 44, Issue 12, 15 August, 2023

View this journal online at wileyonlinelibrary.com

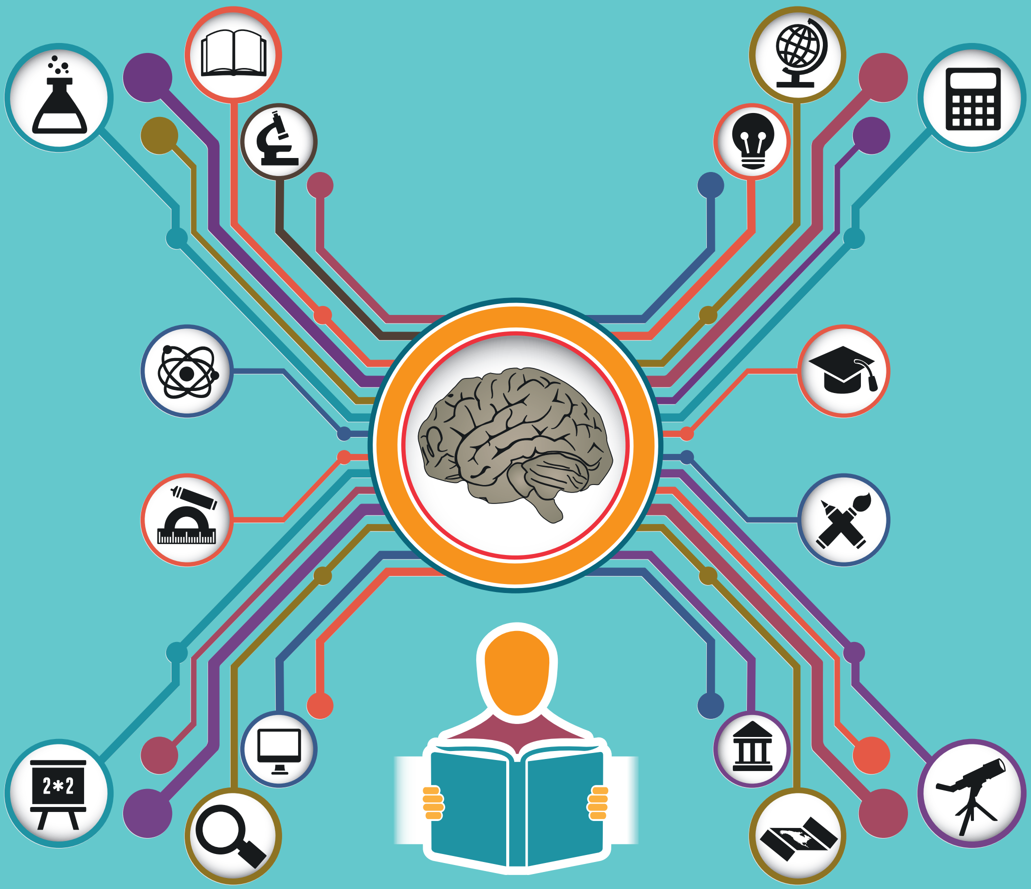

WILEY

ISSN 1097-0193

### HUMAN BRAIN MAPPING

### A Journal Devoted to Functional Neuroanatomy and Neuroimaging

### Editor-in-Chief

**Simon B. Eickhoff**
Institute for Systems Neuroscience
Heinrich-Heine University, Düsseldorf, Germany

### Deputy Editors-in-Chief

**Gary Egan**
Monash Biomedical Imaging
Monash University, Victoria, Australia

**Sophia Frangou**
Icahn School of Medicine
at Mount Sinai, New York, USA

**Martijn van den Heuvel**
Brain Center Rudolf Magnus,
University of Medical Center,
Utrecht, Netherlands

### Associate Editors

**Annabel Chen**
Nanyang Technological University, Singapore

**Michael J. Farrell**
Monash University, Victoria, Australia

**Massimo Filippi**
Università Vita-Salute San Raffaele, Milan,
Italy

**Sophia Frangou**
Icahn School of Medicine at Mount Sinai
New York, USA

**Cheryl L. Grady**
Rotman Research Institute
Toronto, Canada

**Joachim Gross**
Centre for Cognitive Neuroimaging
University of Glasgow, Glasgow, UK

**Martin A. Lindquist**
John Hopkins, Bloomberg School of Public
Health, MD, USA

**Tomáš Paus**
Rotman Research Institute
Toronto, Canada

**Indira Tendolkar**
Radboud University, Nijmegen,
The Netherlands

**Paul Thompson**
Laboratory of Neuro Imaging,
University of Southern California,
Los Angeles, CA, USA

**Helen Zhou**
Yong Loo Lin School of Medicine, National
University of Singapore, Singapore

### Editorial Board

**Susan Bookheimer**
Center for Cognitive Neuroscience
UCLA School of Medicine, USA

**Allen R. Braun**
NIDCD, National Institutes of Health
Bethesda, USA

**Christian Buechel**
Univ-Krankenhaus Eppendorf
Hamburg, Germany

**Marco Catani**
King's College London, UK

**Franco Cauda**
University of Turin, Italy

**Vincent Clark**
University of New Mexico, USA

**Olivier David**
Université Joseph Fourier, France

**Mark D'Esposito**
University of California, Berkeley,
USA

**Julien Doyon**
Functional Neuroimaging Unit
University of Montreal, Canada

**Eugene Duff**
University of Oxford, UK

**Timothy Q. Duong**
Research Imaging Institute,
University of Texas Health Science
Center, USA

**Paul Fletcher**
University of Cambridge, UK

**Jia-Hong Gao**
Brain Research Imaging
Center The University of Chicago,
USA

**David C. Glahn**
Yale University School of Medicine
Hartford, USA

**Gregory S. Hickok**
Center for Cognitive
Neuroscience University of California,
Irvine, USA

**Barry Horwitz**
NIDCD, National Institutes of Health
USA

**Peter Jezzard**
John Radcliffe Hospital
University of Oxford, UK

**David N. Kennedy**
Harvard Medical School, USA

**Neda Ladbon-Bernasconi**
Montreal Neurological Institute
Canada

**Nicholas Lange**
Harvard Medical School, USA

**Tatia M. C. Lee**
State Key Laboratory of Brain &
Cognitive Sciences, The University of
Hong Kong

**Louis Lemieux**
UCL Institute of Neurology, UK

**Bernard Mazoyer**
CNRS/CEA, Université de Bordeaux
France

**Gregory McCarthy**
Yale University, USA

**A. Randy McIntosh**
Rotman Research Institute
Toronto, Canada

**Janine Mendola**
McGill Vision Research
McGill University, Canada

**Thomas Nichols**
University of Warwick, UK

**William Penny**
Wellcome Trust Centre for
Neuroimaging, UCL Institute of
Neurology, UK

**Laurent Petit**
CNRS, Université de Bordeaux,
France

**Russel A. Poldrack**
Stanford Unversity, USA

**Jean-Baptiste Poline**
Imagen Orsay Service Hospitalier
Frédéric Joliot, France

**Stephen Smith**
Center for Functional MRI of the
Brain, University of Oxford, UK

**Elizabeth Sowell**
Children'sHospital Los Angeles, USA

**Li Hai Tan**
Shenzhen Institute of Neuroscience
Shenzhen, P.R. China

**Kate Watkins**
John Radcliffe Hospital
University of Oxford, UK

**Richard G. Wise**
Cardiff University, UK

**Roger Woods**
UCLA School of Medicine, USA

**Mark Woolrich**
Oxford Centre for Human Brain
Activity, University of Oxford, UK

**Jinhu Xiong**
University of Iowa, USA

**Robert Zatorre**
MontrealNeurological Institute, Canada

**Open Access and Copyright**
All articles published by *Human Brain Mapping* submitted after January 1, 2020 are fully open access: immediately freely available to read, download, and share. All articles accepted from August 6, 2019 are published under the terms of a Creative Commons license.
Copyright on any research article published by *Human Brain Mapping* is retained by the author(s).

Further information about open access licenses and copyright can be found at: https://authorservices.wiley.com/open-research/open-access/index.html. Information on the licenses used by *Human Brain Mapping* can be found at: https://onlinelibrary.wiley.com/journal/10970193.

**Disclaimer**
The Publisher and Editors cannot be held responsible for errors or any consequences arising from the use of information contained in this journal; the views and opinions expressed do not necessarily reflect those of the Publisher and Editors, neither does the publication of advertisements constitute any endorsement by the Publisher and Editors of the products advertised. Wiley Open Access articles posted to repositories or websites are without warranty from Wiley of any kind, either express or implied, including, but not limited to, warranties of merchantability, fitness for a particular purpose, or non-infringement. To the fullest extent permitted by law Wiley disclaims all liability for any loss or damage arising out of, or in connection, with the use of or inability to use the content.

### HUMAN BRAIN MAPPING

A Journal Devoted to
Functional Neuroanatomy
and Neuroimaging

VOLUME 44, NUMBER 12, 15 AUGUST, 2023

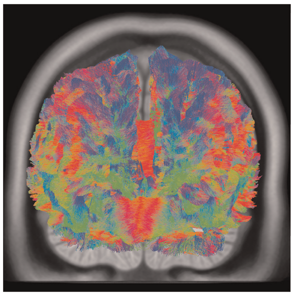

*See article by, Segura-Amil al., pages 4439-4451, this issue*

### CONTENTS

◆ *4426* An automatic interpretable deep learning pipeline for accurate Parkinson's disease diagnosis using quantitative susceptibility mapping and T1-weighted images
**Yida Wang, Naying He, Chunyan Zhang, Youmin Zhang, Chenglong Wang, Pei Huang, Zhijia Jin, Yan Li, Zenghui Cheng, Yu Liu, Xinhui Wang, Chen Chen, Jingliang Cheng, Fangtao Liu, Ewart Mark Haacke, Shengdi Chen, Guang Yang, and Fuhua Yan**
***Published online 19 June 2023***

◆ *4439* Programming of subthalamic nucleus deep brain stimulation with hyperdirect pathway and corticospinal tract-guided parameter suggestions
**Alba Segura-Amil, Andreas Nowacki, Ines Debove, Katrin Petermann, Gerd Tinkhauser, Paul Krack, Claudio Pollo, and T. A. Khoa Nguyen**
***Published online 15 June 2023***

◆ *4452* Pattern classification based on the amygdala does not predict an individual's response to emotional stimuli
**Tim Varkevisser, Elbert Geuze, Max A. van den Boom, Karlijn Kouwer, Jack van Honk, and Remko van Lutterveld**
***Published online 23 June 2023***

◆ *4467* A novel geometry-based analysis of hippocampal morphometry in mesial temporal lobe epilepsy
**Laura Fischbach, Tobias Bauer, Kersten Diers, Juri-Alexander Witt, Mar Brugues, Valeri Borger, Martin Schidlowski, Attila Rácz, Tobias Baumgartner, Randi von Wrede, Daniel Paech, Bernd Weber, Alexander Radbruch, Hartmut Vatter, Albert J. Becker, Hans-Jürgen Huppertz, Christoph Helmstaedter, Rainer Surges, Martin Reuter, and Theodor Rüber**
***Published online 22 June 2023***

(***continued on overleaf***)

(*continued from overleaf*)

◆ *4480* Improved prediction of glioma-related aphasia by diffusion MRI metrics, machine learning, and automated fiber bundle segmentation
**Boshra Shams, Klara Reisch, Peter Vajkoczy, Christoph Lippert, Thomas Picht, and Lucius S. Fekonja**
***Published online 15 June 2023***

◆ *4498* Symptomatic responses elicited by electrical stimulation of the cingulate cortex: Study of a cohort of epileptic patients and literature review
**Yansong Xue, Hao Yan, Guiliang Hao, Ying Gao, Xueyuan Wang, Duanyu Ni, Liang Qiao, Wei Shu, and Tao Yu**
***Published online 15 June 2023***

◆ *4512* Using cortico-cerebellar structural patterns to classify early- and late-trained musicians
**Joseph J. Shenker, Christopher J. Steele, Robert J. Zatorre, and Virginia B. Penhune**
***Published online 16 June 2023***

◆ *4523* Sparsity-guided multiple functional connectivity patterns for classification of schizophrenia via convolutional network
**Renping Yu, Cong Pan, Lingbin Bian, Xuan Fei, Mingming Chen, and Dinggang Shen**
***Published online 15 June 2023***

◆ *4535* One single-person bicycling enhances interpersonal cooperation via increasing interpersonal neural synchronization in left frontal cortex
**Lin Li, Huiling Wang, Yixuan Lin, and Xianchun Li**
***Published online 26 June 2023***

◆ *4545* Neural dissociation between reward and salience prediction errors through the lens of optimistic bias
**Ya Zheng and Shuting Mei**
***Published online 19 June 2023***

◆ *4561* Within-network brain connectivity during a social optimism task is related to personal optimism and optimism for in-group members
**Dominik Andreas Moser, Mihai Dricu, Raviteja Kotikalapudi, Gaelle Eve Doucet, and Tatjana Aue**
***Published online 21 June 2023***

◆ *4572* Negative versus withdrawn maternal behavior: Differential associations with infant gray and white matter during the first 2 years of life
**Karlen Lyons-Ruth, Banu Ahtam, Frances Haofei Li, Sarah Dickerman, Jennifer E. Khoury, Michaela Sisitsky, Yangming Ou, Michelle Bosquet Enlow, Martin H. Teicher, and P. Ellen Grant**
***Published online 7 July 2023***

(*continued on overleaf*)

(*continued from overleaf*)

◆ *4590* Striatonigrostriatal connectivity-based cross-species parcellation of human and macaque substantia nigra
**Xiaoluan Xia, Xinglin Zeng, Fei Gao, Lin Hua, Shaohui Huang, and Zhen Yuan**
***Published online 22 June 2023***

◆ *4605* Widespread intra-axonal signal fraction abnormalities in bipolar disorder from multicompartment diffusion MRI: Sensitivity to diagnosis, association with clinical features and pharmacologic treatment
**Erick Jorge Canales-Rodríguez, Norma Verdolini, Silvia Alonso-Lana, María Llanos Torres, Francesco Panicalli, Isabel Argila-Plaza, Elena Rodriguez-Cano, Irene Montoro, Beatriz Garcia-Ruiz, Esther Jimenez, Cristina Varo, Anna Lluch, Caterina del Mar Bonnin, Silvana Maluf, Marc Pujol, Núria Jaurrieta Guarner, Salvador Sarró, Eduard Vieta, Elisabet Vilella, Raymond Salvador, and Edith Pomarol-Clotet**
***Published online 26 June 2023***

◆ *4623* Hippocampal grading provides higher classification accuracy for those in the AD trajectory than hippocampal volume
**Cassandra Morrison, Mahsa Dadar, Neda Shafiee, D. Louis Collins, and for Alzheimer's Disease Neuroimaging Initiative**
***Published online 26 June 2023***
